# Study on the Preparation and Corrosion–Wear Properties of TiN/Sn Coatings on the Ti-25Nb-3Zr-2Sn-3Mo Titanium Alloy

**DOI:** 10.3390/ma18051160

**Published:** 2025-03-05

**Authors:** Jiang Pu, Yan Dai, Kunmao Li, Li Chen

**Affiliations:** 1 School of Physics and Mechatronic Engineering, Guizhou Minzu University, Guiyang 550025, China; pujiang@gzmu.edu.cn; 2 School of Materials and Architectural Engineering, Guizhou Normal University, Guiyang 550025, China; yyandai@163.com

**Keywords:** Ti-25Nb-3Zr-2Sn-3Mo titanium alloy, TiN/Sn coating, composite-strengthened layer, corrosion–wear, synergy factor

## Abstract

Due to its excellent specific strength, corrosion resistance, and biocompatibility, titanium alloy is often used as a biological implant material. In order to address the issues of low hardness and poor wear resistance of the Ti-25Nb-3Zr-2Sn-3Mo titanium alloy, a TiN/Sn coating with good biocompatibility was deposited on its surface using a new composite modification technology of surface mechanical strengthening + surface mechanical coating. By taking advantage of the wear resistance of TiN and the adhesiveness of Sn, a composite coating with corrosion–wear resistance was formed to improve its corrosion–wear resistance. Using TiN/Sn powders of different ratios (10% Sn, 20% Sn, 30% Sn, and 40% Sn) as media, the alloy was subjected to a combined strengthening treatment of surface mechanical attrition and solid-phase coating under a nitrogen atmosphere. The microstructure and mechanical properties of the composite-strengthened layer were tested by means of XRD, SEM-EDS, a nanoindentation tester, a white-light interferometer, and a reciprocating wear tester. Moreover, the corrosion–wear properties of the samples under different loads and electrochemical conditions were analyzed. The results show that the surface composite-strengthened layer of the alloy consisted of a TiN/Sn coating + a mechanical deformed layer. With an increase in the Sn content, the thickness of the TiN/Sn coating continuously increased, while the thickness of the mechanical deformed layer continuously decreased. The composite-strengthened layer had good comprehensive mechanical properties. In the SBF solution, the corrosion–wear resistance of the composite-strengthened samples improved; the degree of wear first decreased and then increased with the increase in the Sn content, and it reached the optimal value when the Sn content was 30%. Compared with the raw sample, the corrosion of the coating sample increased, but the wear significantly decreased. The corrosion–wear synergy factor κ value first increased and then decreased with the increase in the Sn content, reaching a maximum value at the 20% Sn content. This is the result of the combined effect of the corrosion resistance and wear resistance of the coating.

## 1. Introduction

Due to its excellent specific strength, corrosion resistance, and biocompatibility, titanium alloy is often used as a biological implant material in the knee and hip joints. However, the existence of the synergistic effect of tribocorrosion in the organism can accelerate the degradation of the implant material, leading to the generation of a large amount of wear debris [1]. Eventually, this induces symptoms such as inflammatory reactions and bone resorption [2,3], and the service life of the implant is significantly shortened. Therefore, improving the hardness and wear–corrosion resistance is the key to preventing the failure of the implant. In recent years, researchers have employed techniques such as oxidation [4], chemical vapor deposition [5], mechanical strengthening [6], shot peening [7], and surface heat treatment [8] to strengthen the surface of titanium alloys, aiming to enhance their hardness and wear resistance. Compared with most existing technologies, Surface Mechanical Attrition Treatment (SMAT) is a surface-strengthening technology with advantages such as low cost, high efficiency, and ease of operation. Its principle is to cause severe plastic deformation on the surface through the high-frequency impact of steel balls on the sample; a gradient nanostructure, well bonded to the substrate, is formed on the metal surface, which improves hardness, strength, and wear resistance [9,10,11,12]. Wan Yun [13] and Wang Ronghua et al. [14]. demonstrated that the surface grains of the material are refined after SMAT, which increases its yield stress; however, the SMAT time is highly restricted, and the material’s ductility also decreases. Chen et al. [15] found that the fatigue resistance of an AZ31B alloy was improved when treated using SMAT, but its corrosion resistance decreased. Wen et al. [16] found that the wear resistance of pure titanium treated with SMAT was enhanced, but there was significant material loss and limited ductility. In summary, the drawbacks of SMAT are that the treated samples have relatively high reactivity in air or biological media; this not only reduces their corrosion resistance but also exerts a negative impact on biocompatibility. Moreover, the enhancements in strength and wear resistance are limited.

In this paper, a new composite modification method, Surface Mechanical Composite Strengthening (SMCS) (surface mechanical strengthening + surface mechanical coating), is proposed based on SMAT. The surface of the Ti-25Nb-3Zr-2Sn-3Mo sample is repeatedly impacted and rubbed by high-speed moving grinding balls, causing plastic deformation on the surface layer, which gradually undergoes work hardening. Under the further mechanical action of the grinding balls, dislocations in the surface-layer grains are induced to form sub-grain boundaries or even amorphization through pile-up and entanglement, leading to the refinement and strengthening of the microstructure and forming a strengthened layer with a certain thickness [17,18,19]. Then, taking advantage of the good biocompatibility of TiN [20], by adding a small amount of TiN powder and Sn powder and capitalizing on the cold-welding and mechanical coating effects of Sn on the surface of the titanium alloy when the grinding balls impact at high speed, a composite-strengthened layer consisting of a TiN/Sn coating and a mechanical deformation layer is finally formed. Following this, the microstructure, phase composition, and micro-mechanical properties of the composite-strengthened layer are characterized. To simulate the wear and tear of the Ti-25Nb-3Zr-2Sn-3Mo alloy in practical applications, the corrosion–wear behavior of the composite-strengthened layer under different electrochemical conditions is studied, and the failure mechanism of the composite-strengthened layer under the interaction of chemical, electrochemical, and mechanical factors is discussed.

## 2. Experimental Materials and Methods

### 2.1. Experimental Materials and Coating Preparation Methods

The Ti-25Nb-3Zr-2Sn-3Mo alloy was selected as the experimental material (with the following main components and mass fractions: Ti66.268%, Nb25.5%, Zr3.05%, Sn2.050%, Mo 3.05%). The samples are in the shape of cylinders, with specifications of Ø16 × 8 mm, and were annealed in a vacuum furnace (SKGL-1200, Shanghai Jvjing Precision Instrument Manufacturing Co., Ltd., Shanghai, China) at 800 °C, held at this temperature for 1 h, and then cooled to room temperature along with the furnace. TiN powder (2–3 μm, Hebei Badu Metal Materials Co., Ltd., Shijiazhuang, China) and Sn powder (200 nm, Hebei Jianuo Metal Materials Co., Ltd., Hengshui, China) were used to prepare the TiN/Sn coating.

**Figure 1:**
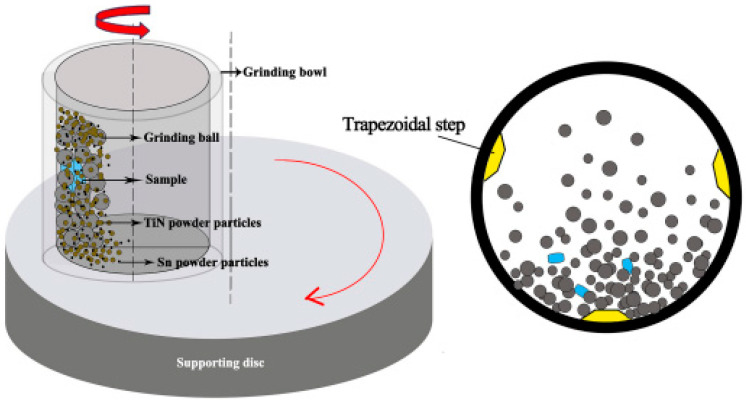
The preparation principle of the TiN/Sn composite coating.

The composite-strengthened layer was prepared using a QM-3SP4 planetary ball-milling mechanism (Nanjing Nanda Instrument Co., Ltd., Nanjing, China). The ball-milling tank is made of stainless steel with a volume of 500 mL. To enhance the impact force between the grinding balls and the samples during the ball-milling process, three trapezoidal steps are welded at different positions on the inner wall of the ball-milling tank. The grinding balls (Zhejiang Yiwu Taizi Steel Ball Co., Ltd., Yiwu, China) are cemented carbide balls of different diameters, which consist of Zro_2_. The rotational speed of the ball milling mechanism is 350 r/min, and the experiment lasts for 8 h. During the experiment, argon gas at a pressure of 0.05 MPa was filled into the tank to prevent the coating from oxidizing. TiN/Sn composite coatings were prepared with 10 g of powders of different ratios (TiN-10%Sn, TiN-20%Sn, TiN-30%Sn, TiN-40%Sn). During the ball-milling process, the powder gradually deposits onto the surface of the substrate under the impact of the grinding balls, and the continuous and dense coating is gradually formed. The preparation principle of the TiN/Sn composite coating is shown in Figure 1.

### 2.2. Characterization of Microstructure Morphology

Under the dark-field shooting mode, a metallographic microscope (OM, Leica DMI8, Leica Microsystems, Wetzlar, Germany) was used to photograph the cross-sectional microstructure after corrosion with Kroll solution (100 mL of distilled water + 1–3 mL of HF + 2–6 mL of HNO_3_), and the metallographic dark-field photos were observed. The surface, cross-section, and wear scar morphology of the sample were observed using a Zeiss field-emission scanning electron microscope (SEM, Zeiss Merlin Compact, Hitachi, Ltd., Tokyo, Japan). The composition and elemental distribution of the sample were analyzed using an energy-dispersive spectroscope (EDS, Oxford Xplore-30, Oxford Instruments, Abingdon, UK) equipped with a scanning electron microscope. The morphology of secondary electron SEM observations and EDS were performed at an operating voltage of 20 kV and a spot size of 60 μm. The phase composition of the composite coatings was characterized using an X-ray diffractometer (XRD, X’Pert PRO, PANalytical, Ltd., Almelo, The Netherlands) with Cu-K α radiation. The surface wear morphology and roughness were collected using a white light interferometer (3D, Shenzhen Zhongtu Instrument Co., Ltd., Shenzhen, China) at 20 times the lens, and the three-dimensional morphology construction and analysis were performed in the equipped software of XtremeVision-Pro 1.2.

### 2.3. Micro-Mechanical Property Testing

The surface and cross-sectional hardness of the specimens were tested using an MHV-2.0 automatic Vickers hardness tester (Dongguan Guangcai Measurement and Control Technology Co., Ltd., Dongguan, China). The load was 0.245 N, and the load was unloaded after a holding time of 15 s. The hardness and elastic modulus were measured and calculated using a German Bruker Hysitron TI980 (Bruker Corporation, Billerica, MA, USA) nanoindentation instrument. The indentation positions were selected as the surface coating area, the subsurface deformation layer area, and the substrate area. The maximum loading load was 30 mN, the loading rate was 60 mN/min, the unloading rate was 60 mN/min, and the holding time was 5 s.

### 2.4. Corrosion–Wear Performance Test

The corrosion–wear behavior of the alloy was studied using a self-made corrosion–wear testing machine [20] in the laboratory. The corrosive solution was SBF solution (Sinopharm Chemical Reagent Co., Ltd., Shanghai, China, with the following main components: Na^+^: 142.0, K^+^: 5.0, Mg^2+^: 1.5, Ca^2+^: 2.5, Cl^−^: 147.8, HCO^3−^: 4.2, HPO_4_^−^: 1.0, SO_4_^2−^: 0.5) at a temperature of 36.5 ± 0.5 °C. The reciprocating mode was adopted during the friction process, and the friction pair was an Al_2_O_3_ ball with a diameter of 7 mm. During the friction wear test, the sliding speed was set at 15 mm/s; the normal loads were set at 2 N and 4 N, respectively; and the one-way sliding distance was 12 mm. The electrochemical tests were carried out using a traditional three-electrode system, namely the working electrode (the tested material), the platinum electrode as the counter electrode, and the saturated calomel electrode (SCE) as the reference electrode. The tribocorrosion tests were carried out under three different flow electrochemical conditions: (1) open circuit potential (OCP), (2) applied cathodic potential (−1.5 V), and (3) applied anodic potential (+0.5 V). The samples were immersed in SBF solution for 3000 s before the tests. The tests were divided into three stages: before wear (10 min), during wear (30 min), and after wear (20 min). The changes in the coefficient of friction as well as the potential–current responses throughout the wear process were measured and recorded. After wear, an optical 3D profilometer and a scanning electron microscope were used to measure the wear volume and wear morphology of the different samples under various electrochemical conditions and loads. To reduce test errors, the wear volume was taken as the average of three tests.

## 3. Results and Discussion

### 3.1. Phase Composition of Coating

**Figure 2:**
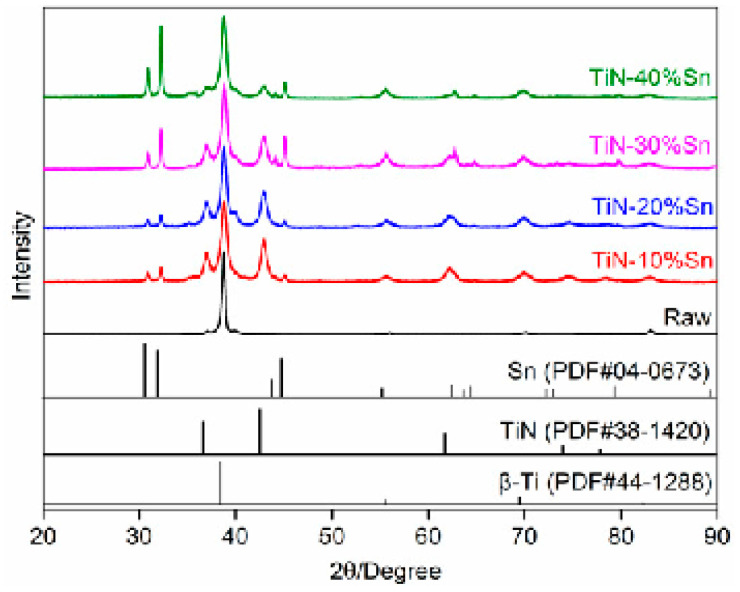
XRD of the samples before and after SMCS.

The X-ray diffraction patterns of the surface of the Ti-25Nb-3Zr-2Sn-3Mo alloy before and after SMCS are shown in Figure 2. The coating mainly consists of three phases, Ti, TiN, and Sn, indicating that no alloying occurred during the mechanical ball-milling process. As the proportion of Sn powder increases, the intensity of the diffraction peaks of Sn gradually increases, while the intensity of the diffraction peaks of TiN shows a downward trend. The X-ray diffraction peaks of the sample have broadened significantly after SMCS; the reason for this is that the intense impact of the grinding balls on the sample surface and powder particles causes severe plastic deformation during the ball-milling process, resulting in lattice distortion and the grain refinement of the alloy matrix, TiN, and Sn [21].

### 3.2. Coating Microstructure

**Figure 3:**
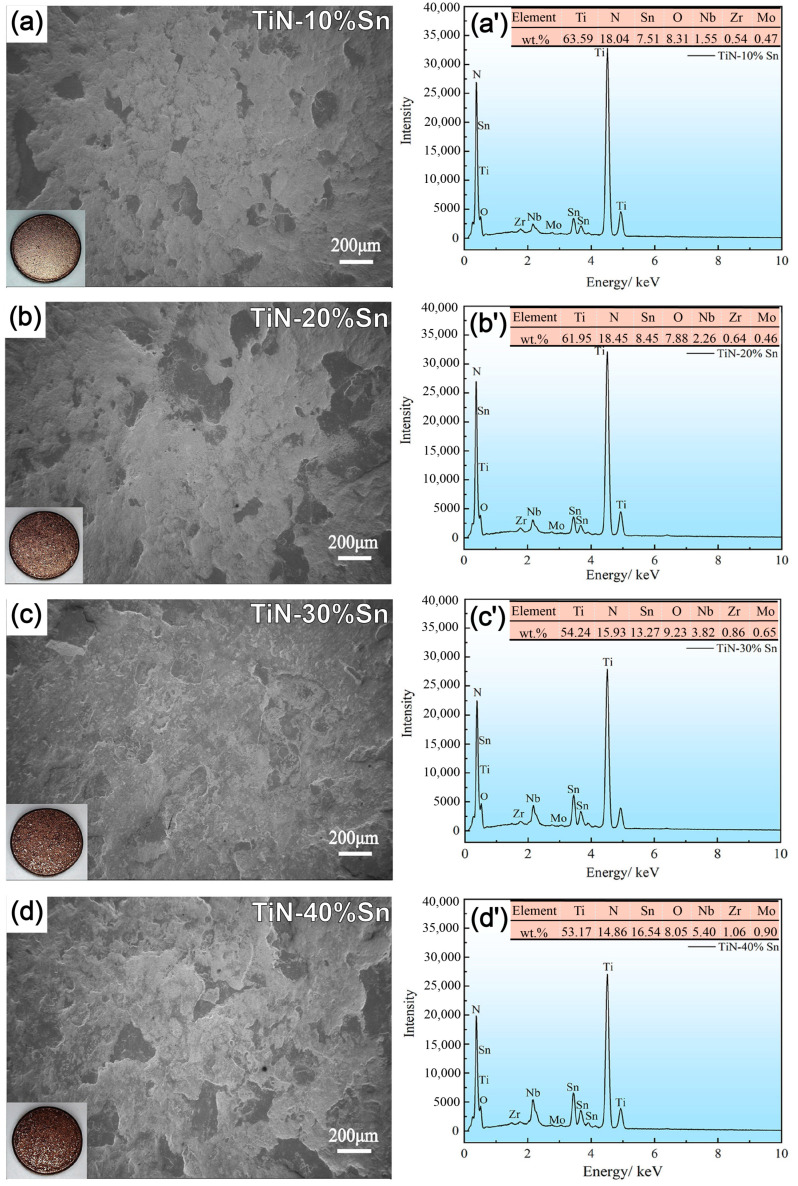
Surface SEM and EDS morphologies of the TiN/Sn coating: (**a**,**a’**) 10% Sn; (**b**,**b’**) 20% Sn; (**c**,**c’**) 30% Sn; (**d**,**d’**) 40% Sn.

The surface SEM and EDS morphologies of the TiN/Sn coating are shown in Figure 3. The inset in the lower-left corner is the macroscopic morphology. The alloy substrate is basically covered by the coating after SMCS. The intensity of the energy spectrum of Sn continuously increases with the increase in the proportion of Sn powder. During the SMCS process, the formation of the coating is attributed to the cold-welding between the powder particles and the surface-layer metal [4]. On the one hand, under the repeated action of the grinding balls, the TiN powder and Sn powder particles gradually adhere; finally, a relatively continuous extruded-composite coating is formed on the substrate surface [4]. On the other hand, the formed coating undergoes a dynamic cycle of deformation, spalling, and cold-welding under the impact of the grinding balls. The oxidation of the coating was controlled due to the protection of the atmosphere, and the internal cohesion of the coating was maintained [11]. Eventually, a coating with a rough surface and a dense interior was formed. The roughness of the coating continued to increase with the increase in the amount of Sn, and the plastic powder accumulated continuously [11].

**Figure 4:**
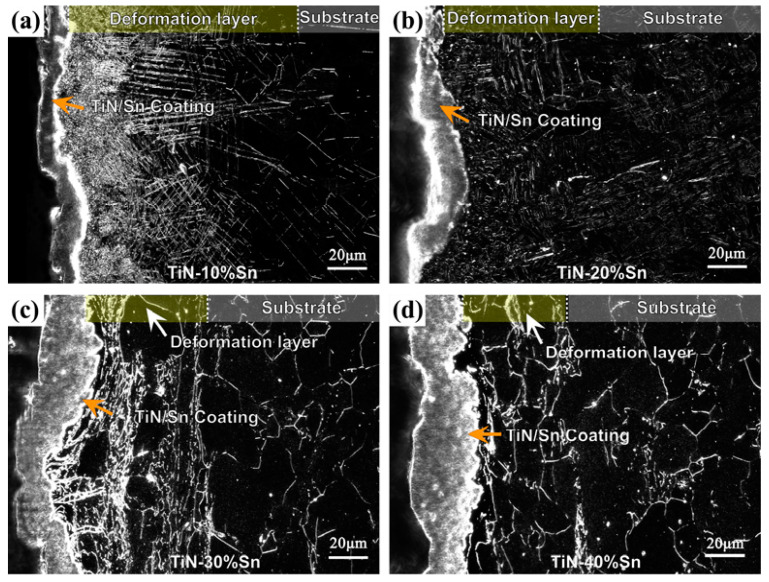
Metallographic dark-field photograph of the cross-section of coating samples after SMCS: (**a**) 10% Sn; (**b**) 20% Sn; (**c**) 30% Sn; (**d**) 40% Sn.

The metallographic structures of the cross-sections of samples with different powder proportions under dark-field conditions are shown in Figure 4. Each sample surface had an obvious coating area after the SMCS, and the coating thickness increased with the increase in the proportion of Sn powder. In addition, obvious deformation band microstructure characteristics appeared on the substrate surface layer, with these characteristics gradually weakening with the increase in depth, presenting a continuously changing gradient structure. The reason is that the substrate surface layer undergoes continuous plastic deformation due to the intense mechanical impact of the grinding balls, ultimately resulting in the formation of deformation bands and grain refinement [22]. Contrary to the trend of coating thickness change, the thickness of the deformed layer decreases as the Sn amount increases. For the 10% Sn sample, severe plastic deformation occurred on the surface layer, with very significant characteristics of deformation bands and grain refinement. However, for the 40% Sn sample, there was almost no plastic deformation on the surface layer, the grain size was relatively large, and the shape was close to equiaxed. The reason for this is that it is easier for the Sn powder to adhere to and be deposited on the substrate surface layer and form a coating with the increase in the proportion of Sn powder [10]. At the same time, the coating thickens rapidly, cushioning the direct collision between the substrate and the grinding balls. Eventually, the Ti-25Nb-3Zr-2Sn-3Mo alloy forms a composite-strengthened layer consisting of a surface TiN/Sn coating + a subsurface mechanical deformation layer.

**Figure 5:**
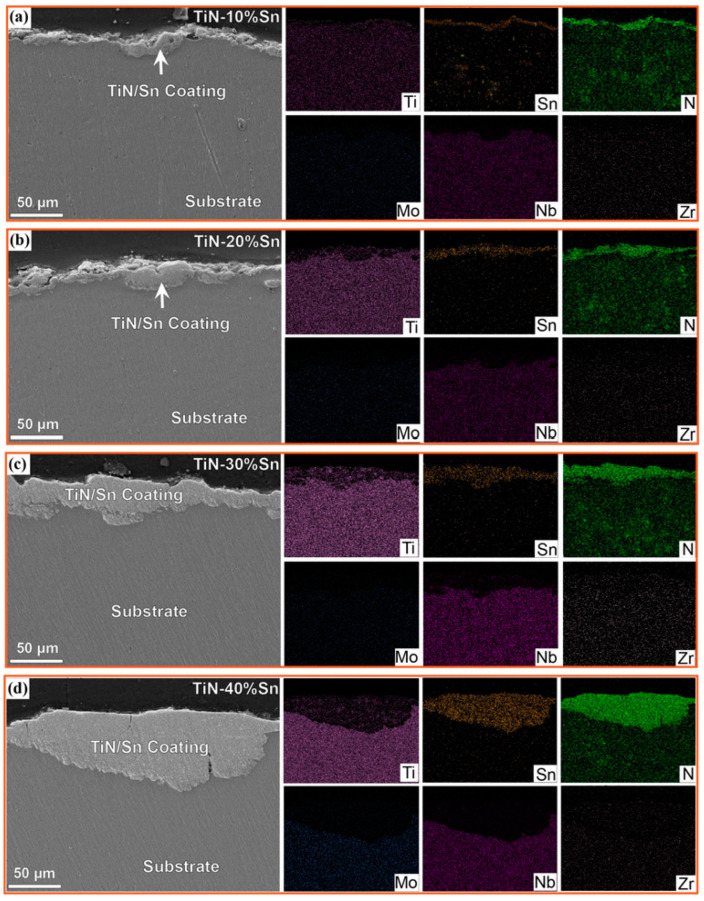
Cross-section morphology and mapping of coating samples after SMCS: (**a**) 10% Sn; (**b**) 20% Sn; (**c**) 30% Sn; (**d**) 40% Sn.

The SEM morphology and energy spectrum of the coating cross-section are shown in Figure 5. The interfaces of the TiN/Sn coatings with different powder proportions generally exhibit a winding and tortuous feature. The phenomena of impact and extrusion into the substrate surface layer are visible in some areas [4]. No pores or looseness are observed on the coating or its interface. This is because the substrate is relatively soft, and the repeated collisions between the grinding balls and the substrate cause plastic deformation on the alloy surface layer in the initial stage of ball-milling [10]. The hard TiN particles are directly squeezed into the titanium substrate under the impact of the grinding balls, forming a surface-particle-strengthened layer. With the increase in the ball-milling time, the activity of the powder increases, and cold-welding occurs between the powder and the substrate. Under the action of the grinding balls, the powder gradually deposits and densifies, ultimately forming a TiN/Sn coating of a certain thickness. The mapping analysis showed the continuous increase in the coating thickness as the amount of SN increased, which is consistent with the results in Figure 4. Under the field of view of the optical microscope, the thickness ranges and average thicknesses of the coatings for samples with 10%, 20%, 30%, and 40% Sn are 5.45–13.34 μm, 5.40–16.84 μm, 9.78–28.90 μm, 10.86–32.38 μm and 8.08 μm, 14.05 μm, 18.33 μm, and 23.55 μm, respectively. As a plastic material, Sn increases the overall adhesion of the powder, raising the probability of cold-welding during the impact of the grinding balls. Meanwhile, the particle radius of Sn powder is significantly smaller than that of TiN powder, endowing it with a stronger packing ability [23]. Therefore, the increase in the amount of Sn promotes the increase in the coating thickness.

### 3.3. Nanoindentation Results

**Figure 6:**
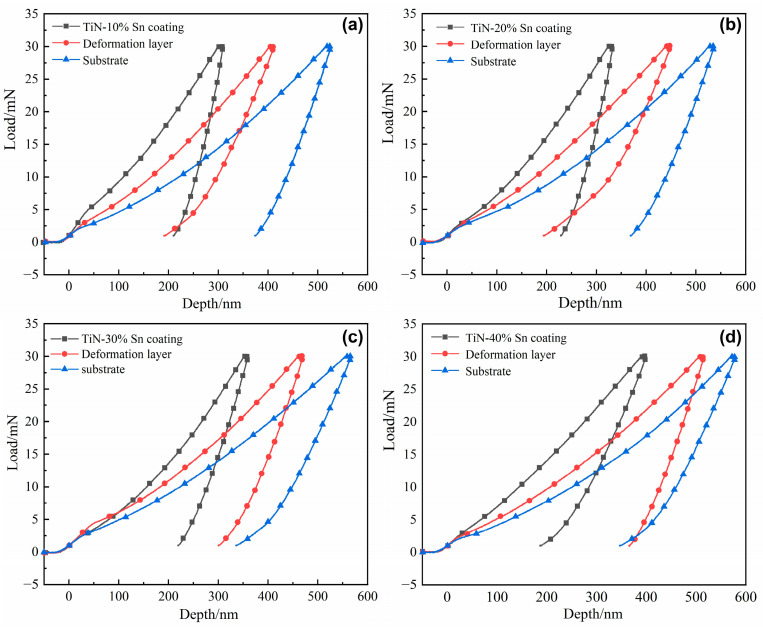
Nanoindentation load-displacement curves for the cross-section of samples after SMCS: (**a**) 10%Sn, (**b**) 20%Sn, (**c**) 30%Sn, (**d**) 40%Sn.

Nanoindentation tests were carried out on the cross-section of the Ti-25Nb-3Zr-Sn-3Mo samples. The test positions were selected as the surface coating area, the subsurface mechanical deformation layer area, and the substrate (at a position more than 200 μm away from the surface layer). The load was increased from 0 to 30 mN. The obtained load–displacement curves are shown in Figure 6, and the maximum indentation depth H_max_, nano-hardness H, and elastic modulus E are listed in Table 1. The H_max_ gradually increased from the surface to the interior after SMCS. For the same region of different samples, H_max_ increased with the increase in the proportion of Sn powder. It can be seen that the TiN/Sn particle-strengthened coating improves the hardness of the surface layer of the titanium alloy, and with the increase in the proportion of Sn powder, the coating plasticity is improved, and hardness is reduced. The H_max_ of the subsurface mechanical deformation layer exhibits the same regularity. The cushioning effect provided by the thicker coating reduces the degree of deformation of the substrate, thus reducing the work-hardening effect. The values of H and E calculated from the indentation depth are listed in Table 1. It can be seen that the H and E values of the 10% Sn coating are 9.50 GPa and 221.20 GPa, respectively, which are 2.98 times and 2.28 times higher than those of the substrate. Research shows that the higher the H/E value of a material, the better its wear resistance; the higher the H^3^/E^2^ value, the better the material’s ability to resist plastic deformation [20,24]. When the proportion of Sn powder is relatively low (10% Sn, 20% Sn), the values of H/E and H^3^/E^2^ are highest in the mechanical deformation layer, followed by those in the coating area, and when the proportion of Sn powder is relatively high (30% Sn, 40% Sn), the values of H/E and H^3^/E^2^ are highest in the coating area. This indicates that the composite-strengthened layer has good load-bearing capacity and plastic deformation resistance [25].

### 3.4. Corrosion–Wear Performance and Analysis

#### 3.4.1. OCP and COF During the Friction Process

**Figure 7:**
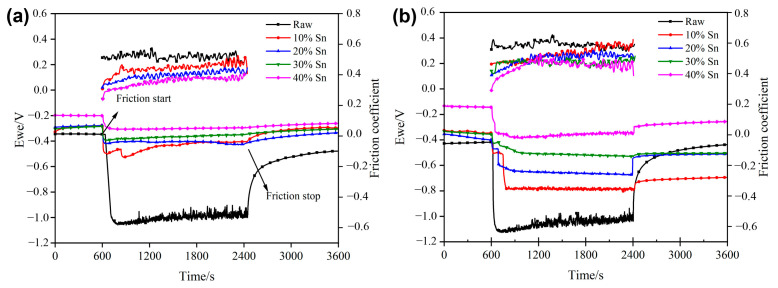
Time-COF and time-OCP curves for samples in SBF solution after SMCS under different loads: (**a**) COF and OCP (2 N); (**b**) COF and OCP (4 N).

The Open-Circuit Potential (OCP) and Coefficient Of Friction (COF) curves of the TiN/Sn composite coating changing with time during sliding wear in the SBF solution are shown in Figure 7. The OCP of both the raw sample and the composite-coating sample remains stable in a static state, and the potentials of the composite-coating samples are higher than that of the raw one. After the wear begins, the OCP of the raw sample drops rapidly; subsequently, the potential rises slowly over time. The destruction and repassivation of the oxide film on the surface of the titanium alloy during the wear process may be the main reasons for the change in its potential [26]. The OCP of the coated samples also decreased after the wear began, but the decrease was significantly less than that of the raw samples. This indicates that the coating itself has good corrosion resistance, so even if the surface oxide film is damaged, the potential will not drop significantly. In addition, the formation of the composite coating also improved the wear resistance of the alloy, reducing the degree of surface damage. With the increase in the load (Figure 7b), the magnitude of potential drop in most samples also increases, especially for the 10% Sn and 40% Sn samples, indicating that the increase in load leads to an enhanced promoting effect of wear on corrosion. However, the load has almost no effect on the OCP of the 30% Sn sample, and the potential decrease is minimal, indicating that, from a thermodynamic perspective, the impact of wear on the corrosion tendency is the least. After the wear–corrosion test, the potential of the sample increased slowly, which means that the passive film in the worn area was gradually recovering [27].

The COF of the composite-coated samples is lower than that of the raw samples under both loads. Due to the interaction between corrosion and wear, the COF of the samples increased with the increase in the load [4]: the COF of the raw sample increased from 0.53 under a 2 N load to 0.61 under a 4 N load. The COF showed a decreasing trend as the amount of Sn increased for the composite-coated samples, indicating that Sn can play a certain lubricating role [23]. The COF increased slowly over time and gradually stabilized. The surface roughness of the coating samples was relatively high, the Al_2_O_3_ ball only came into direct contact with the surface protrusions during the initial stage of wear, the protrusions were gradually worn down as the wear progressed, and the sample entered a stage of steady-state wear. The decrease in the COF following SMCS can be ascribed to the TiN/Sn composite coating. The formation of this composite coating boosts the hardness of the alloy, thereby strengthening its ability to resist deformation. The hard TiN phase plays a load-bearing role during the wear process, while the soft Sn phase can act as a lubricating phase, effectively reducing the COF of the alloy.

#### 3.4.2. Influence of Externally Applied Potential

**Figure 8:**
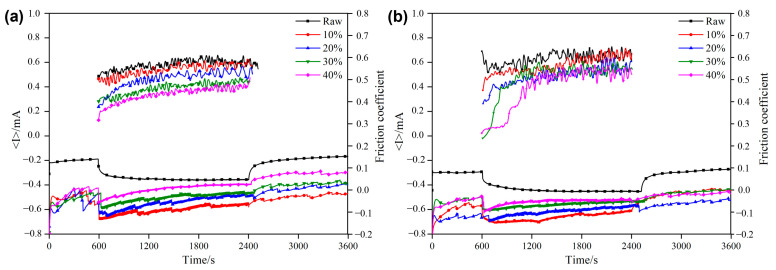
Time-current and time-COF curves for the samples with cathodic protection (−1.5 V) applied under different loads: (**a**) 2 N; (**b**) 4 N.

**Figure 9:**
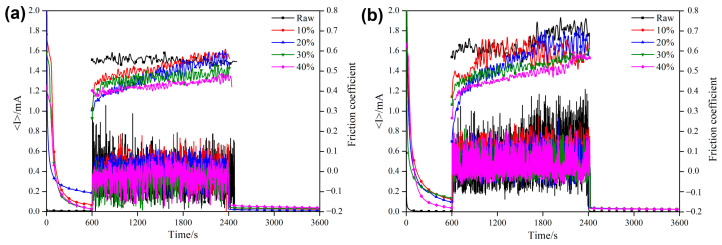
Time-current and time-COF curves for samples with anodic corrosion (+0.5 V) applied under different loads: (**a**) 2 N; (**b**) 4 N.

The changes in the COF and current of the raw sample and the composite coating sample during the wear–corrosion test under the applied potentials of −1.5 V and +0.5 V are shown in Figure 8 and Figure 9, respectively.

The sample was completely electrochemically protected by the applied cathodic potential (*E* = −1.5 V). The measured negative current corresponds to the reduction reactions of dissolving oxygen and water [28]. During the wear process, the currents in both the raw samples and the coating samples decreased with the increase in the load, which may have been caused by the destruction of the surface oxide film during the wear process. During the wear process, the current of the raw sample steadily increased over time, whereas that of the composite-strengthened sample decreased, which may be due to the gradual expansion in the area of the wear scar region of the raw sample as the wear progressed. Meanwhile, the rest of its surface rapidly entered a fully passivated state, and the disruption of the surface passive film resulted in a continuous increase in the current [29]. During the entire test stage, the surface of the coating sample was in a slow passivation state in the SBF solution, and the current gradually decreased. At the moment when wear started, the current increased in a step-like manner due to the appearance of a fresh worn surface; however, the entire surface was still in the passivation stage, so the current continued to decline slowly. Due to the slow wear, the wear scar area was also passivated slowly, resulting in an insignificant current change at the moment when the wear ended [30].

Under different loading conditions, the COF of all samples was higher than that under the corresponding OCP conditions (as shown in Figure 7). The main reason for this could be that the wear debris cannot be dissolved under cathodic protection, resulting in a relatively high roughness in the wear scar area. The COF of the coating samples was lower than that of the raw samples and decreased with the increase in the Sn content, further confirming the lubricating effect of Sn [23]. The gradually increasing wear debris adhered to the wear scar area during the friction process, increasing the frictional resistance and leading to an increase in the COF [4].

With the application of anodic potential (*E* = +0.5 V), as shown in Figure 9, the current increased abruptly at the beginning of wear, indicating that the corrosion resistance of the worn area on the sample surface deteriorated. After the wear stopped, a passive film formed on the sample surface again, and the current rapidly returned to its initial state. These phenomena are consistent with the findings in the existing literature [31]. In addition, the current increases with the increase in the load during the wear process, which may be due to the fact that the area of the wear scar expands with the increase in load [32]. Compared with the OCP condition, the COF of both the raw samples and the coating samples with applied anodic potential also exhibited an increase, possibly attributed to the fact that the anodic potential accelerates the damage in the wear scar area, thereby enlarging the contact area between the grinding ball and the samples [20]. Meanwhile, the accelerated destruction of the surface passive film also weakens its lubricating effect. In addition, the COF of the coating samples is lower than that of the raw samples, indicating a better anti-friction effect. The anodic currents of different samples are shown in Table 2. The static current *I_C_* of the raw sample prior to wear is one order of magnitude lower than that of the coating sample, suggesting that the passive film of the raw sample exhibits better corrosion resistance. However, the *I_W_* of the raw sample during the wear process is significantly higher than that of the coating sample, which may be attributed to the excellent wear resistance of the composite coating [31]. In the SBF solution, even after the application of anodic potential, the destructive effect of wear on the samples is always greater than the corrosion effect. Therefore, under the combined action of corrosion and wear, the corrosion current of the composite-strengthened samples is lower than that of the raw samples. In addition, as the load increases, the *I*_w_ of both the raw samples and the composite-strengthened samples increases, which is likewise due to the enlargement of the wear scar area.

#### 3.4.3. Corrosion–Wear Morphology and Wear Volumes

**Figure 10:**
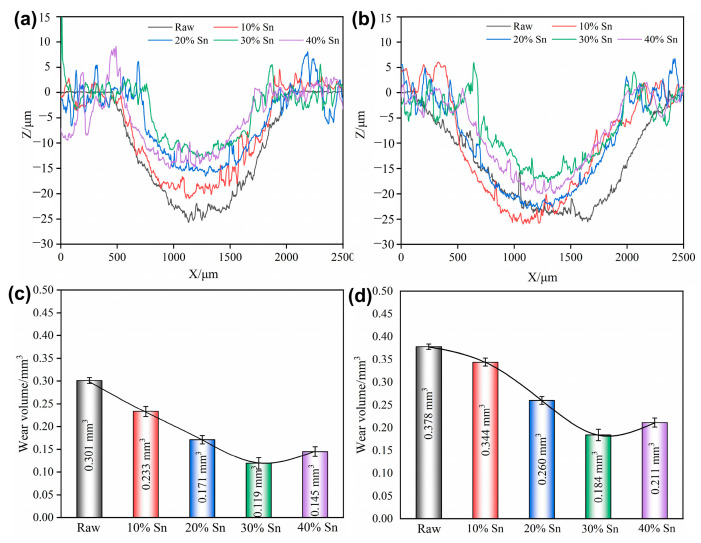
Wear scar profiles and wear volume loss of samples under OCP under different loads: (**a**,**c**) 2 N; (**b**,**d**) 4 N.

**Figure A1:**
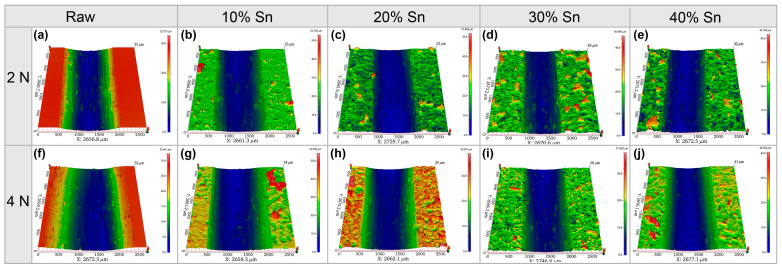
3D wear scar morphologies of samples under OCP under different loads: ((**a**)—2 N, (**f**)—4 N Raw), ((**b**)—2 N, (**g**)—4 N 10% Sn), ((**c**)—2 N, (**h**)—4 N 20% Sn), ((**d**)—2 N, (**i**)—4 N 30% Sn), (**e**)—2 N, (**j**)—4 N 40% Sn).

The wear scar profile and wear volumes of the raw samples and composite-strengthened samples after the tribocorrosion test under OCP are shown in Figure 10 and Figure A1. A large number of parallel grooves appear along the sliding direction on the raw sample, and the width and depth of the wear scar increase from 1565 μm and 23.86 μm under the 2 N load to 2256 μm and 25.41 μm under the 4 N load, respectively. The degree of wear of the coating samples first decreases and then increases with the increase in the Sn content; among them, the sample with 30% Sn powder has the smallest wear scar width (1093 μm under the 2 N load and 1319 μm under the 4 N load) and the shallowest depth (13.26 μm under the 2 N load and 18.89 μm under the 4 N load). The wear volume loss of the coating samples is reduced compared to that of the raw samples, indicating that the wear resistance of the alloy can be improved through SMCS. The formation of the composite-strengthened layer enhances the alloy’s hardness and its resistance to plastic deformation, and it can effectively reduce the material volume loss caused by pure mechanical wear [33].

**Figure 11:**
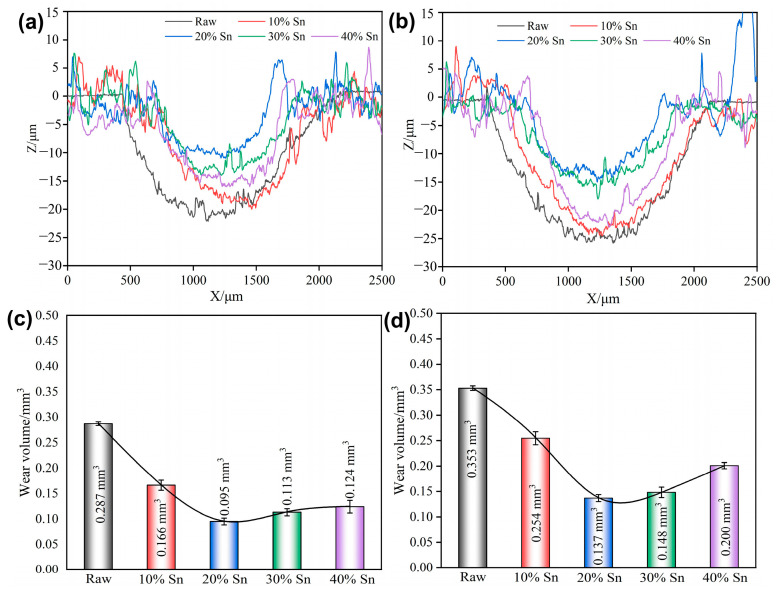
Wear scar profiles and wear volume loss of samples with cathodic potential (*E* = −1.5 V) applied under different loads: (**a**,**c**) 2 N; (**b**,**d**) 4 N.

**Figure A2:**
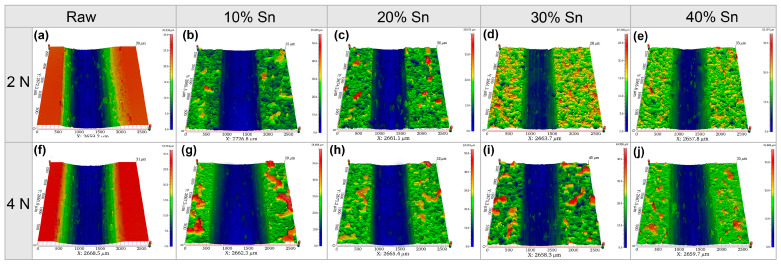
3D wear scar morphologies of samples with cathodic potential (*E* = −1.5 *V*) applied under different loads: ((**a**)—2 N, (**f**)—4 N Raw), ((**b**)—2 N, (**g**)—4 N 10% Sn), ((**c**)—2 N, (**h**)—4 N 20% Sn), ((**d**)—2 N, (**i**)—4 N 30% Sn), (**e**)—2 N, (**j**)—4 N 40% Sn).

The wear scar profile and wear volumes of the raw samples and composite-strengthened samples after the tribocorrosion test with applied cathodic potential (*E* = −1.5 V) are shown in Figure 11 and Figure A2. Compared with the OCP, the width and depth of the wear scars of the raw samples and the composite-strengthened samples decrease under cathodic protection. This is because only the wear effect, and not the corrosion effect, exists under cathodic protection [34]. The wear volume loss of the samples before and after SMCS under different loads increases overall with the increase in the load. The coating samples under cathodic protection exhibit the same regularity as under OCP; that is, the wear volume loss first decreases and then increases with the increase in the Sn content. The difference is that the coating sample with 20% Sn content has the least material loss under cathodic protection. On the one hand, this is because the coating with 20% Sn has a higher hardness and better wear resistance. Under cathodic protection, since only the wear effect exists, a harder coating is better able to resist plastic deformation. On the other hand, it also indicates that the coating with 20% Sn has poor corrosion resistance, and the corrosion has a stronger promoting effect on wear.

**Figure 12:**
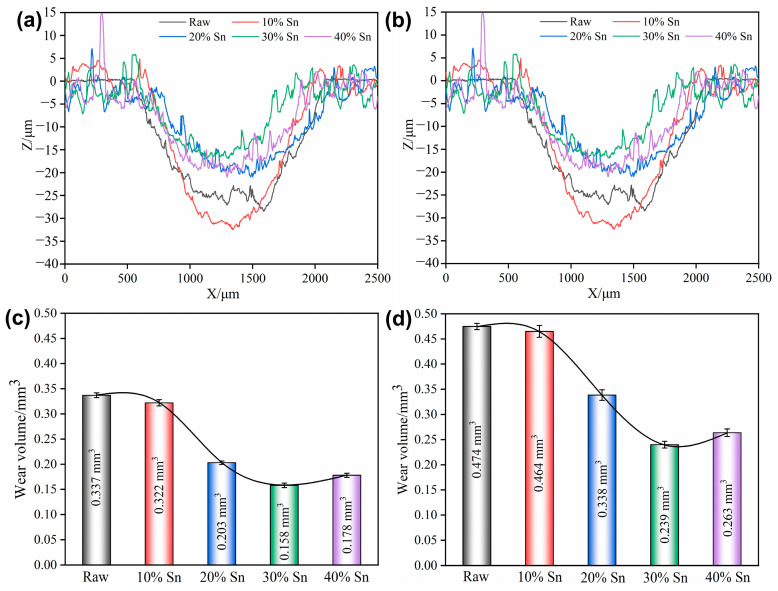
Wear scar profiles and wear volume loss of samples with anodic potential (*E* = +0.5 V) applied under different loads: (**a**,**c**) 2 N; (**b**,**d**) 4 N.

**Figure A3:**
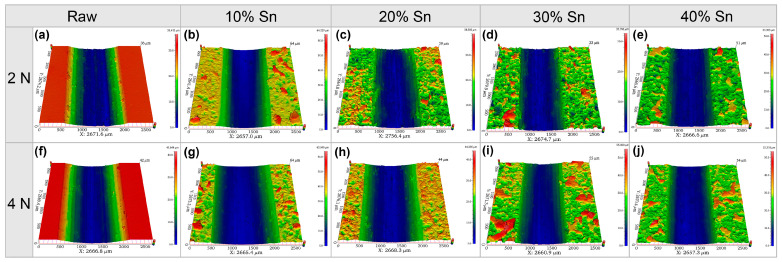
3D wear scar morphologies of samples with anodic potential (*E* = +0.5 *V*) applied under different loads. ((**a**)—2 N, (**f**)—4 N Raw), ((**b**)—2 N, (**g**)—4 N 10% Sn), ((**c**)—2 N, (**h**)—4 N 20% Sn), ((**d**)—2 N, (**i**)—4 N 30% Sn), ((e)—2 N, (**j**)—4 N 40% Sn).

The wear scar profile and wear volume loss of the raw samples and composite-strengthened samples after the tribocorrosion test with applied anodic potential (*E* = +0.5 V) are shown in Figure 12 and Figure A3. Compared with the samples under OCP or those under applied cathodic potential, the width and depth of the wear scars of the raw samples and composite-strengthened samples increase under applied anodic potential, and the increase in the composite-strengthened samples is more significant. This is because the composite-strengthened layer itself has poor corrosion resistance, its corrosion rate accelerates under anodic potential, and the promoting effect of corrosion on wear is also enhanced [20]. The wear scar width of both the raw samples and the composite-strengthened samples also increases with the increase in the load. Similar to the OCP, the sample with 30% Sn has the minimum wear scar depth and the lowest wear volume loss. The wear scar surfaces of both the raw samples and the composite-strengthened samples are smoother under applied anodic potential, and the wear debris can be rapidly dissolved anodically once it is generated. The smooth wear scar surface helps reduce the COF; however, under anodic potential, it is difficult for an oxide layer to form in the wear scar area, which weakens the lubrication effect. Additionally, the increase in the depth and width of the wear scar also increases the contact area between the grinding ball and the surface, thus increasing friction. Under the combined influence, the COF shows no significant difference compared to that under the protection of cathodic potential.

#### 3.4.4. Synergistic Effect of Corrosion and Wear Under Applied Anodic Potential

When material undergoes wear in a corrosive environment, the total volume loss *V_T_* can be quantitatively analyzed using the following expression [35]:

(1) $$V_{T}=V_{C}+V_{M}+\Delta V_{S}$$

where *V_C_* is the volume loss due to corrosion in the absence of wear, *V_M_* is the volume loss due to pure mechanical wear, and ∆*V_S_* is the volume loss due to the synergistic action of corrosion and wear. It consists of two items:

(2) $$\Delta V_{S}=V_{MC}+V_{CM}$$

*V_MC_* is the effect of wear on corrosion, and *V_CM_* is the effect of corrosion on wear.

*V_C_* and *V_MC_* are obtained using Faraday’s law [36].

(3) $$V_{C}=\frac{M\cdot I_{C}\cdot t}{n\cdot F\cdot \rho }$$

(4) $$V_{MC}=\frac{M\int_{600}^{2400}I_{W}dt}{n\cdot F\cdot \rho }$$

where *M* is the relative atomic mass of the titanium alloy (47.867 g/mol), *I_C_* is the average corrosion current, *I_W_* is the average wear current, *t* is the corrosion–wear time (3600 s), *n* is the oxidation valence in reaction, *F* is the Faraday constant (96,500 C/mol), and ρ is the density of the titanium alloy (4.5 g/cm^3^).

Combining Equations (1)–(4), *V_CM_* is obtained using the following expression:

(5) $$V_{CM}=V_{T}-V_{C}-V_{M}-V_{MC}$$

**Figure 13:**
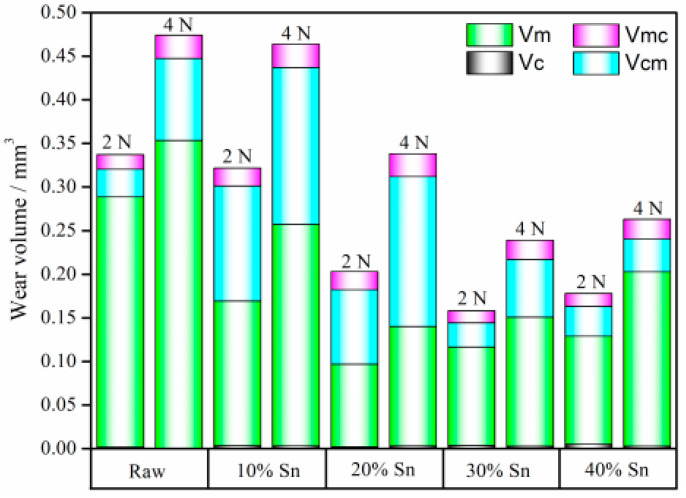
Corrosion–wear components of the samples with applied anodic potential under different loads.

**Figure 14:**
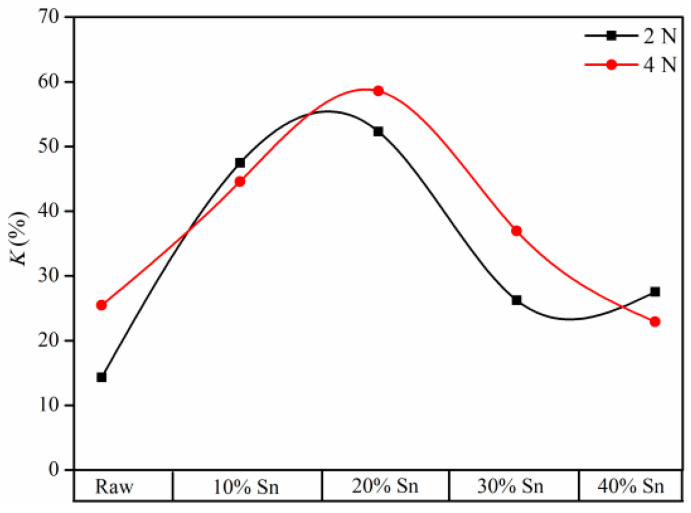
*κ* curves of the samples under different loads.

The corrosion–wear components of the raw samples and composite-strengthened samples with applied anodic potential (+0.5 V) under different loads are shown in Figure 13 and Table A1. Compared with the raw samples, the *V_C_* of the coating samples increases, while its *V_M_* decreases significantly. Under loads of 2 N and 4 N, respectively, the volume loss contributed by the *V_M_* of the raw samples accounts for 85% and 74% of the *V_T_*, while the volume loss contributed by *V_C_* only accounts for 0.51% and 0.069% of the *V_T_*. The change in ∆*V_S_* is more complex, as ∆*V_S_/V_T_* is defined as the synergy factor *κ*. As shown in Table A1 and Figure 14, the value of *κ* first increases and then decreases with the increase in the Sn content under 2 N and 4 N, reaching the maximum value at the Sn content of 20%. As mentioned previously, when the Sn content is low, the composite-strengthened layer has a relatively high hardness. However, the coating is thin, and its corrosion resistance is poor, therefore the promoting effect of corrosion on wear is relatively strong [37]. For the sample with 10% Sn, due to its very thin coating and relatively poor wear resistance, the *κ* value is not the highest. The sample with 20% Sn has better wear resistance but poor corrosion resistance, resulting in a relatively high △*V_S_*; therefore, the κ value of this sample is the highest. As the Sn content increases, although the hardness of the coating decreases to some extent, its thickness and corrosion resistance increase simultaneously. Consequently, the κ value drops rapidly and reaches its minimum when the Sn content is 30%. Overall, the *κ* value of the coating samples is higher than that of the raw samples. For relatively thin coating samples, the rough surface structure and voids in the coating provide diffusion channels for corrosive media, forming a galvanic corrosion cell between the coating and the substrate [38,39]. Similarly, during the wear–corrosion process, the spalled hard TiN coating forms hard abrasive particles under the action of the grinding ball, causing three-body abrasive wear, which further increases the *V_MC_*, and the synergistic effect generated by the interaction between corrosion and wear accelerates the material volume loss.

## 4. Conclusions

(1) With the increase in the Sn content, the average thickness and roughness of the composite coating increase, while the thickness of the mechanical deformation layer decreases. The hardness of the composite coating decreases with the increase in the Sn content, but it is higher than that of the substrate. The elastic modulus and nano-hardness of the coating area are significantly greater than those of the substrate. The scratch penetration depth under variable loads for the 20% Sn and 30% Sn samples is only one-third of that of the raw sample, indicating good comprehensive mechanical properties.
(2) The OCP values of the composite-strengthened samples are higher than those of the raw samples and increase with the increase in the Sn content. Wear leads to a decrease in the OCP, and the decrease amplitude of the potential increases with the increase in the load. Under anodic corrosion, the corrosion currents of the composite-strengthened samples are higher than those of the raw samples, while the wear currents are lower than those of the raw samples. Moreover, the corrosion current decreases with the increase in the Sn content, and the wear current first decreases and then increases. The 30% Sn sample has the lowest wear current.
(3) Under different loads and electrochemical conditions, the wear volume loss of the composite-strengthened layer is lower than that of the raw sample, and the wear volume loss first decreases and then increases with the increase in the Sn content. Under cathodic protection, the 20% Sn sample has the lowest wear volume loss under both 2 N and 4 N loads. Under OCP and anodic corrosion, the 30% Sn sample exhibits the minimum wear volume loss.
(4) For both the raw samples and the composite-strengthened samples, the volume loss caused by pure mechanical wear plays a dominant role during the corrosion–wear process. Compared with the raw samples, the pure corrosion *V_C_* of the coating samples increases, while the pure wear *V_M_* decreases significantly. The κ values first increase and then decrease with the increase in the Sn content, reaching a maximum value at a Sn content of 20%. This is the result of the combined effects of the corrosion resistance and wear resistance of the coating.

## Figures and Tables

**Table 1:** Indentation mechanical properties at different positions of the cross-section of samples after SMCS.

Sample	Position	H_max_/nm	Elastic Modulus, E/GPa	Nano-Hardness, H/GPa	H/E × 10^−2^	(H^3^/E^2^)/zzzzGPa × 10^−2^
10% Sn	Coating	309.65	221.20	9.50	4.29	1.75
	Deformation layer	412.092	91.47	7.61	8.32	5.27
	Substrate	546.48	74.93	4.15	5.54	1.27
20% Sn	Coating	333.13	200.83	8.49	4.23	1.52
	Deformation layer	449.48	88.22	6.20	7.03	3.06
	Substrate	556.62	68.39	4.08	5.96	1.45
30% Sn	Coating	359.92	132.73	8.51	6.41	3.50
	Deformation layer	470.64	92.36	5.36	5.80	1.80
	Substrate	567.13	63.59	4.03	6.34	1.62
40% Sn	Coating	399.77	92.34	8.19	8.87	6.44
	Deformation layer	516.50	92.36	4.39	4.75	0.99
	Substrate	579.57	62.02	3.87	6.24	1.50

**Table 2:** Anodic currents of samples under different loads.

Load/N	*I*/A	Raw	10% Sn	20% Sn	30% Sn	40% Sn
2	*I* *C*	(1.72 ± 0.30) × 10^−5^	(3.20 ± 0.32) × 10^−5^	(1.81 ± 0.19) × 10^−5^	(3.55 ± 0.29) × 10^−5^	(5.01 ± 0.60) × 10^−5^
	*I* *W*	(3.35 ± 1.37) × 10^−4^	(4.14 ± 0.69) × 10^−4^	(4.17 ± 0.56) × 10^−4^	(2.79 ± 0.76) × 10^−4^	(3.03 ± 0.84) × 10^−4^
4	*I* *C*	(3.28 ± 1.90) × 10^−6^	(3.06 ± 0.30) × 10^−5^	(3.03 ± 0.43) × 10^−5^	(2.71 ± 0.15) × 10^−5^	(2.76 ± 0.20) × 10^−5^
	*I* *W*	(5.37 ± 1.80) × 10^−4^	(5.40 ± 0.88) × 10^−4^	(5.23 ± 0.81) × 10^−4^	(4.45 ± 0.85) × 10^−4^	(4.55 ± 0.96) × 10^−4^

## Data Availability

The original contributions presented in this study are included in the article. Further inquiries can be directed to the corresponding author.

## Appendix A

**Table A1:** Corrosion–wear components of the samples with applied anodic potential under different loads.

Load	Sample	*V_T_*/(mm^3^)	*V_M_*/(mm^3^)	*V_C_*/(mm^3^)	*V_MC_*/(mm^3^)	*V_CM_*/(mm^3^)	∆*V_S_*/(mm^3^)	*κ*%
2 N	Raw	3.37 × 10^−1^	2.87 × 10^−1^	1.71 × 10^−3^	1.66 × 10^−2^	3.17 × 10^−2^	4.83 × 10^−2^	14.33
	10% Sn	3.22 × 10^−1^	1.66 × 10^−1^	3.17 × 10^−3^	2.05 × 10^−2^	1.32 × 10^−1^	1.53 × 10^−1^	47.46
	20% Sn	2.03 × 10^−1^	9.50 × 10^−2^	1.80 × 10^−3^	2.07 × 10^−2^	8.55 × 10^−2^	1.06 × 10^−1^	52.32
	30% Sn	1.58 × 10^−1^	1.13 × 10^−1^	3.52 × 10^−3^	1.38 × 10^−2^	2.77 × 10^−2^	4.15 × 10^−2^	26.25
	40% Sn	1.78 × 10^−1^	1.24 × 10^−1^	5.00 × 10^−3^	1.50 × 10^−2^	3.40 × 10^−2^	4.90 × 10^−2^	27.53
4 N	Raw	4.74 × 10^−1^	3.53 × 10^−1^	3.25 × 10^−4^	2.66 × 10^−2^	9.41 × 10^−2^	1.21 × 10^−1^	25.46
	10% Sn	4.64 × 10^−1^	2.54 × 10^−1^	3.04 × 10^−3^	2.68 × 10^−2^	1.80 × 10^−1^	2.07 × 10^−1^	44.60
	20% Sn	3.38 × 10^−1^	1.37 × 10^−1^	3.01 × 10^−3^	2.60 × 10^−2^	1.72 × 10^−1^	1.98 × 10^−1^	58.58
	30% Sn	2.39 × 10^−1^	1.48 × 10^−1^	2.69 × 10^−3^	2.21 × 10^−2^	6.62 × 10^−2^	8.83 × 10^−2^	36.95
	40% Sn	2.63 × 10^−1^	2.00 × 10^−1^	2.74 × 10^−3^	2.26 × 10^−2^	3.77 × 10^−2^	6.03 × 10^−2^	22.91
